# Immediate Root Fillings

**Published:** 1890-08

**Authors:** M. S. Merchant

**Affiliations:** GIDDINGS, TEXAS.


ARTICLE VI.

### IMMEDIATE ROOT FILLINGS.

BY DR. M. S. MERCHANT, of GIDDINGS, TEXAS.

**Read before the Texas Dental Assosiaion, at Belton, Texas, May 6, 1890.**

It is with some degree of trepidation that I mention my subject, which is "Immediate Root Filling." This operation has been written and talked about so much that we are all tired of it. Still the fact that roots of teeth must be filled, is forced upon us every day; therefore it behooves us to learn how best to do it. To begin: A patient comes to you with an upper central incisor, with large cavity approximating the other central. The tooth is badly discolored, and upon examination you find the pulp is dead and giving off a very offensive odor. You wish to restore the organ to its former beauty and usefulness as nearly as possible. What will do it and how shall it be accomplished? I will give you my method: First, with chisels, excavators and engine burs I remove all decayed dentine and friable enamel, and make a good clear opening into the nerve chamber and canal; then if necessary, with Gates' Glidden drills, open the canal until I can introduce Donaldson's canal cleansers and clean out all the debris; then with small syringe with gold point, I throw hydrogen peroxide into the canal and keep it up until it ceases to foam, when I put on the dam and with Japanese bib. Paper cones made by rolling small bits between the thumb and finger, dry out the canal as well as possible, after which I dip a small bit of the paper into alcohol and force into the canal to assist in extracting the water from the dental tubuli, and dry out as before. Now the hot air syringe is brought into service, and heated air is pumped into the canal; then heat a wire to redness and pass into the canal repeatedly, if necessary, until it is thoroughly dried out. Now with whalebone point, force a solution of gutta percha in chloroform into the canal. Give the chloroform a little time to evaporate; then force a gutta percha cone into the root very firmly and you have almost a perfect if not a perfect root filling. I do not hesitate to do this if there is an abscess, knowing that I have removed the cause, i. e., the dead pulp. And in addition to this have sealed permanently the fountain from whence the poison came that caused it, viz.: the foramen at the apex of the root. The only disinfectant and antiseptic that I have used having been hydrogen peroxide, it having broken up the pus and killed the bugs (if there be any bugs) and made the canal as nearly antiseptic as anything known to me can do it. The abscess will then get well of itself. I of course prefer having a fistulous opening to an abscess, but proceed just the same if there is not; and out of more than one hundred cases I have had to drill through only one process to relieve the pain, and this gave almost immediate relief. In this case the abscess was throbbing and hurting at the time I was filling the root.

Peroxide of hydrogen is a colorless fluid and in decomposing it is said to give off four hundred and seventy-five times its volume of oxygen. When it comes in contact with pus in an abscess it breaks it up and when it has an opening forces it out mechanically. I have used it in a case of abscess of antrum, in which case I was assisted by Dr. L. C. Johnson, the patient's family physician. It was a case of long standing. All the teeth on the right side above were decayed and broken off to the gum. All of them were badly abscessed and the antrum had became involved, the palatine bone on the right side had become soft and there was a large fistulous opening at palatine suture and another opposite first bicuspid. I extracted the teeth and we washed out the antrum twice a week for a short time, the openings healed up, the bones hardened; and in a few months I made her a plate which she is still wearing and she has a healthy mouth. Dr. Friedricks, of New Orleans; when at Galveston last year said dentists are coming here to the Southern Dental Association and making assertions that they are doing "this and that," but he wanted data. It struck me forcibly enough to cause me when I got home to get me some books to keep a record of my work in, and bring with me my book of root filling giving only a record of what I have done since that time, and anyone who wishes to do so may look over it, and see the names of the parties, and where they live, etc. I see some of them every day, and all of them are in easy reach at any time. Now I have not tried to bring up anything new or that is unknown to any well-informed dentist, but there are some of you who are sceptical on the subject of immediate root filling, and the sceptic as a rule is the man who has not given it a fair trial. I only ask you to give it a test trial before you condemn it, and in trying it be thorough or you will make a failure. If you operate thoroughly and intelligently, my word for it you will be successful in saving the tooth, and at the same time save your patient the money and trouble of repeated visits to the dentist.—*Texas Dental Journal.*

---

ARTICLE VII.

